# Three-dimensional band structure and surface electron accumulation of rs-Cd_*x*_Zn_*1*−*x*_O studied by angle-resolved photoemission spectroscopy

**DOI:** 10.1038/s41598-019-44423-9

**Published:** 2019-05-29

**Authors:** Kazutoshi Takahashi, Masaki Imamura, Jang Hyo Chang, Tooru Tanaka, Katsuhiko Saito, Qixin Guo, Kin Man Yu, Wladek Walukiewicz

**Affiliations:** 1 Synchrotron Light Application Center, Saga University, Saga, 840-8502 Japan; 2 Department of Electrical and Electronic Engineering, Saga University, Saga, 840-8502 Japan; 3 Department of Physics, City University of Hong Kong, Kowloon, Hong Kong; 4 Materials Sciences Division, Lawrence Berkeley National Laboratory, Berkeley, California 94720 USA; 5 Department of Materials Science and Engineering, University of California at Berkeley, Berkeley, CA 94720 USA

**Keywords:** Electronic properties and materials, Solar cells, Surfaces, interfaces and thin films

## Abstract

Three-dimensional band structure of rock-salt (rs) Cd_*x*_Zn_*1*−*x*_O (*x* = 1.0, 0.83, and 0.60) have been determined by angle-resolved photoemission spectroscopy (ARPES) using synchrotron radiation. Valence-band features shift to higher binding energy with Zn content, while the conduction band position does not depend strongly on Zn content. An increase of the indirect band gap with Zn-doping is larger than that of the direct band gap, reflecting a weaker hybridization between Zn 3*d* and O 2*p* than that between Cd 4*d* and O 2*p*. Two-dimensional electronic states due to the quantization along surface normal direction are formed in the surface accumulation layer and show non-parabolic dispersions. Binding energy of the quantized two-dimensional state is well reproduced using an accumulation potential with the observed surface band bending and the characteristic width of about 30 Å.

## Introduction

Transparent conducting oxides (TCOs) possess concurrent optical transparency and high electrical conductivity in a single material. These intriguing properties of TCOs are particularly well suited for a wide range of applications including solar cells, displays, light emitting diodes, and transparent electronics^1–3^. CdO shows an inclination for n-type conduction with electron concentration exceeding 10^21^ cm^−3^ and with an electron mobility of higher than 100 cm^2^/Vs, providing a semiconductor material with the conductivity higher than 10^4^ S/cm and transparent in a broad spectral range from IR to visible region^4^. In addition, it has been reported that alloying CdO with ZnO can increase the optical band gap from 2.5 eV to 2.8 eV for the Zn content of 0.25 without significantly affecting the carrier concentration and mobility^5^. Thus the Cd-rich CdZnO alloy is potentially one of the most promising transparent conductors for use in photovoltaics.

The band gap and electrical conductivity are key properties for applications of the material as transparent conducting layer. However, the high degeneracy of the electron gas in CdO and CdZnO and the associated Burstein-Moss (B-M) effect lead to experimental uncertainty over the true value of the intrinsic band gap, for the carrier densities exceeding 10^19^ cm^−3 ^^5,6^. In addition, the electron effective mass in the partially filled conduction band varies with carrier concentration, which complicates the analysis of optical and transport data. In contrast to the optical or transport studies, photoemission and x-ray spectroscopies give more direct insights into their electronic structure. Locations of the valence band maximum and partially filled conduction band have been reported by combined experiment of photoemission and electron-energy-loss spectroscopies^7^. The O 2p partial density of states and shallow core-level hybridization has been revealed by soft x-ray emission and absorption^8^. The widening of bandgap with increasing Zn content in CdZnO alloy has also been reported by Detert *et al*.^9^. However, the exact determination of the direct and indirect gap still seems difficult due to the broadened leading edge of the obtained spectra. Another important aspect of the CdO based semiconductors is the formation of electron accumulation layer at the surface, which is caused by the defect-induced pinning of the surface Fermi energy at the Fermi level stabilization energy, *E*_FS_^10,11^, or the charge neutrality level^12–15^. Formation of quantized electron states has been reported in the surface electron accumulation region of CdO^16,17^.

From the theoretical point of view, a hybridization of O 2*p* state with deeper-lying *d* state of the metal plays an important role in the electronic structure of post-transition metal oxides^18–20^. The *p*-*d* hybridization in the rock-salt (rs) structure with octahedrally-coordinated metal cation pushes the valence-band states to lower binding energies. This anisotropic hybridization vanishes at zone center and produces maxima in the O 2*p* valence bands located at the *L* point and midway along *Γ*-*K* line, and the minimum of conduction band at *Γ* point. The strength of *p*-*d* hybridization depends on the energy difference and spatial extent of both states. Thus, it is important to elucidate the evolution of the three-dimensional band structure with Zn content for whole Brillouin zone (BZ).

In this work, we have performed an angle-resolved photoemission spectroscopy (ARPES) on well-ordered single-crystal thin-film of rs-phase Cd_x_Zn_1−x_O alloys with *x* = 1.0, 0.83 and 0.60 in order to investigate their three-dimensional band structure. We have found that the changes of the indirect and direct band gap are mainly related to the shift of the valence band whereas the charge neutrality level in conduction band shows small shift. The in-plane dispersion of quantized sub-band states in the electron accumulation region at the surface reveals non-parabolic dispersion of the conduction band. Thickness of the charge accumulation layer and the surface carrier density has been estimated from the binding energy of quantized sub-band states.

## Experiment

The rs-Cd_*x*_Zn_1−*x*_O thin films were epitaxially grown on MgO(001) substrates by a molecular-beam epitaxy (MBE) system with a radio frequency radical cell as the source of oxygen. The base pressure of the growth chamber was less than 6 × 10^−8^ Pa. 7N Zn and 6N Cd were used as source materials. Prior to the films growth, MgO substrates were ultrasonically cleaned in organic solvents. Then, rs-Cd_*x*_Zn_1−*x*_O films with the thickness of 100 nm were grown at a substrate temperature of 250 °C and a growth rate of approximately 1 nm/min. During the growth, a reflection high-energy electron diffraction (RHEED) pattern was monitored to characterize the crystalline quality. In this study, the Cd flux ratio *f* (= [Cd]/([Zn] + [Cd])) was changed between 0.94 and 1.0 to obtain rs-Cd_*x*_Zn_1−*x*_O thin films with different Cd composition, and three rs-Cd_*x*_Zn_1−*x*_O thin films with *x* = 1.0, 0.83, and 0.60 measured by energy dispersive x-ray spectroscopy (EDX) were prepared.

Photoemission measurements were performed at the beamline BL13 in Saga Light Source^21^. Prior to the photoemission measurements, samples were annealed at 300 °C in ultra-high vacuum for about 14 hours to remove surface contaminations. Surface cleanliness and the structure have been characterized by core-level photoemission and low-energy electron diffraction (LEED) measurements. Sharp (1 × 1) LEED spots were observed on *x* = 1.0 and 0.83 samples. The sample with *x* = 0.60 showed LEED patterns with broad and weak spots indicating slightly poor crystalline quality. This is consistent with X-ray diffraction (XRD) data obtained from this sample which show a broadened diffraction peak. Core-level photoemission spectra were measured using a photon energy of 700 eV. Valence band photoemission spectra were measured using photon energies between 14 eV and 130 eV. The Fermi energy and the energy resolution were confirmed by measurements for the Fermi level of the gold reference. The overall energy resolutions were estimated to be 0.40 and 0.04 eV for core-level and valence band measurements, respectively.

## Results and Discussion

**Figure 1:**
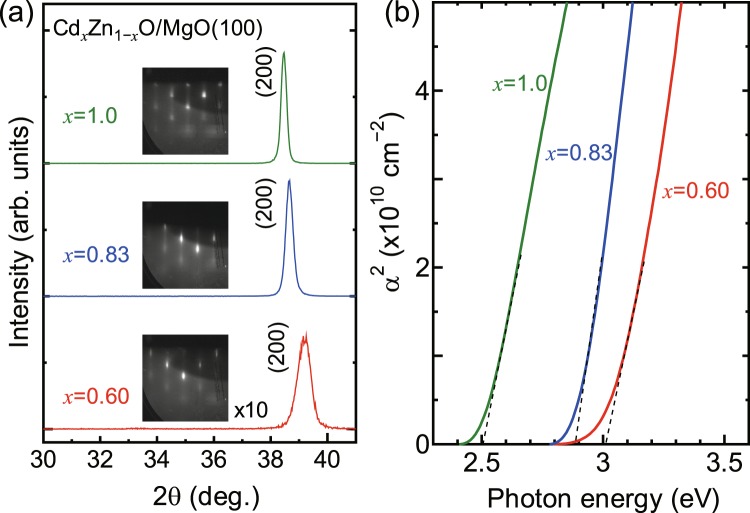
(**a**) X-ray diffraction profiles of Cd_*x*_Zn_1−*x*_O films with *x* = 1.0, 0.83, and 0.60. The insets show reflection high-energy electron diffraction patterns of the films. (**b**) Square plots of optical absorption coefficients (α) of Cd_*x*_Zn_1−*x*_O films with *x* = 1.0, 0.83, and 0.60.

X-ray diffraction profiles of Cd_*x*_Zn_1−*x*_O films with *x* = 1.0, 0.83, and 0.60 are shown in Fig. 1a. A diffraction peak from (200) plane of rs-Cd_*x*_Zn_1−*x*_O is found for all films, and the diffraction angle shifted to higher angle side with decreasing Cd content *x* due to the replacement of Cd site by smaller Zn atoms. The full width at half maximum (FWHM) of the diffraction peak becomes wide at *x* = 0.6, indicating the deterioration of the crystallinity although the single crystal epitaxial layer was obtained as confirmed by a reflection high-energy electron diffraction pattern shown in the inset of Fig. 1a. The optical band gap energies of Cd_*x*_Zn_1−*x*_O with *x* = 1.0, 0.83, and 0.60 were determined as 2.5, 2.88, and 3.0 eV, respectively, from the square plots of optical absorption coefficients (α) obtained from optical transmission and reflection as shown in Fig. 1b. The electrical properties were characterized by Hall effect measurements using the van der Pauw configuration at room temperature. The resistivity (*ρ*), electron concentration (*n*), and mobility (*μ*) of Cd_*x*_Zn_1−*x*_O are summarized in Table 1. Since the electron concentration are high as in the order of 10^20^ cm^−3^, the optical band gap energy obtained above is expected to be influenced by Burstein-Moss (B-M) shift^5^. The detailed study on the structural, optical, and electrical properties of Cd_*x*_Zn_1−*x*_O epitaxial layers on MgO (100) substrates will be published elsewhere.

**Table 1:** Electrical properties of Cd_*x*_Zn_1−*x*_O films with *x* = 1.0, 0.83, and 0.60.

*x*	*ρ* (Ωcm)	*n* (cm^−3^)	*μ* (cm^2^/Vs)
1.0	6.0 × 10^−4^	2.3 × 10^20^	45.2
0.83	1.9 × 10^−4^	5.2 × 10^20^	62.1
0.60	4.2 × 10^−4^	6.1 × 10^20^	24.8

**Figure 2:**
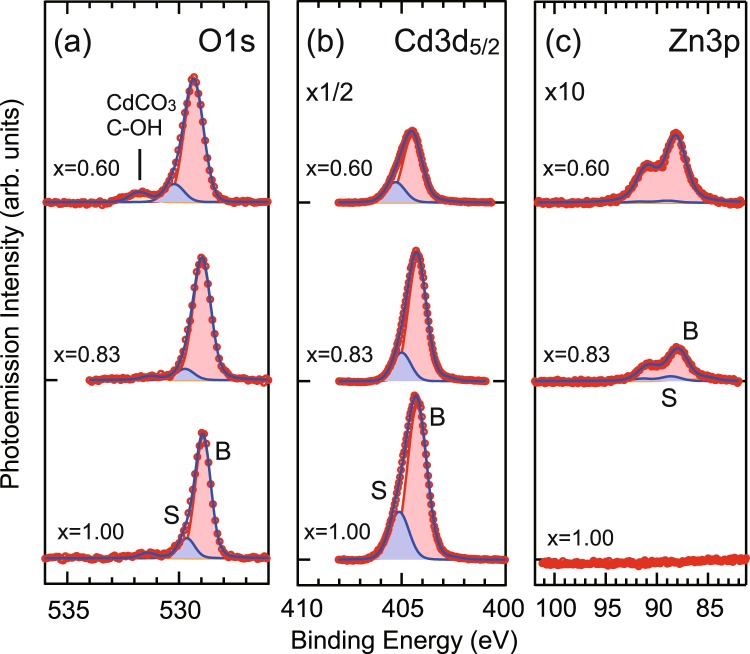
O 1*s*, Cd 3*d*_5/2_, and Zn 3*p* core-level spectra measured at the photon energy of 700 eV. Red and blue components from the least squares fitting correspond to the bulk and surface components, respectively.

Cd 3d_5/2_, Zn 3p and O 1s core-level spectra of Cd_*x*_Zn_1−*x*_O with *x* = 1.0, 0.83, and 0.60 are shown in Fig. 2. The spectra are well fitted using Voigt shape peaks. Besides the main peak (red), an additional component (blue) originating from the surface layer with downward band bending was found for all Cd_*x*_Zn_1−*x*_O samples. Similar shifted-component from the surface atoms has been reported on InAs surface with downward band bending forming an electron accumulation layer^16^. The bulk component of the O 1*s* occurs at binding energies of 528.95 ± 0.01, 528.98 ± 0.01, and 529.33 ± 0.01 eV for the Cd content of *x* = 1.0, 0.83, and 0.60, respectively. The component at about 531.6 eV for *x* = 0.60 sample originates from residual surface contamination of CdCO_3_ and C-OH species as reported previously^22^. The bulk component of Cd3*d*_5/2_ occurs at binding energies of 404.26 ± 0.01, 404.24 ± 0.01, and 404.48 ± 0.01 eV for the Cd content of *x* = 1.0, 0.83, and 0.60, respectively. The Zn 3*p* spectra were fitted by two spin-orbit split 3*p* components with Δ*E*_SO_ = 3.0 eV^23,24^. The bulk component of Zn 3*p*_3/2_ occurs at the binding energies of 87.74 ± 0.05 and 88.13 ± 0.05 eV for the Cd content of *x* = 0.83, and 0.60, respectively. The shifts of the surface components relative to the bulk components were fitted assuming the same value for all core levels. The downward band bendings have been estimated as 0.87 ± 0.01, 0.76 ± 0.01, and 0.82 ± 0.01 eV, for *x* = 1.00, 0.83, and 0.60, respectively. Thus, quantized conduction sub-bands are expected in such deep wells at the surface.

**Figure 3:**
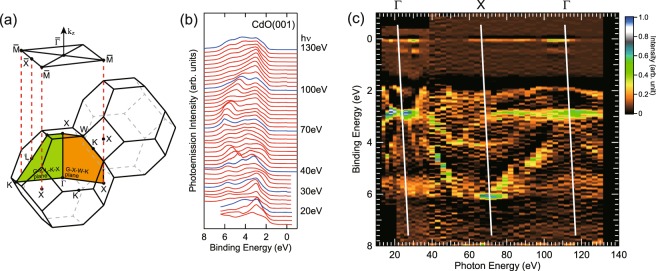
(**a**) The three-dimensional BZ and (001)-projected surface BZ of rs-CdO. (**b**) The EDCs of CdO (*x* = 1.0) at normal emission measured with photon energy ranging between 14 and 130 eV. (**c**) The second derivative image of the EDCs shown in (**b**). Vertical lines indicate the high symmetry points along surface normal direction.

Figure 3a shows the three-dimensional BZ and (001)-projected surface BZ of rs-CdO. Figure 3b shows energy distribution curves (EDCs) of CdO (*x* = 1.0) at normal emission measured with photon energy ranging between 14 eV and 130 eV. The overall character of the curves is similar to those reported previously^16^. The second derivative images of the curves are shown in Fig. 3c. The normal emission spectra show periodic structures corresponding to the band dispersion along surface normal (*Γ*-*X*) direction. Prominent features are found at around 2.8 eV in binding energy measured with photon energies of about 26 and 118 eV. Similarly, another prominent feature is found at 6 eV in binding energy with photon energy of 70 eV. The points of high symmetry along surface normal direction are indicated by white lines. These points follow the free electron dispersion with an inner potential of U_0_ = −8.8 eV and an effective mass of 0.88*m*_e_. Also, two dispersing bands along *Γ*-*X*-*Γ* points are noticed in the ARPES mapping.

**Figure 4:**
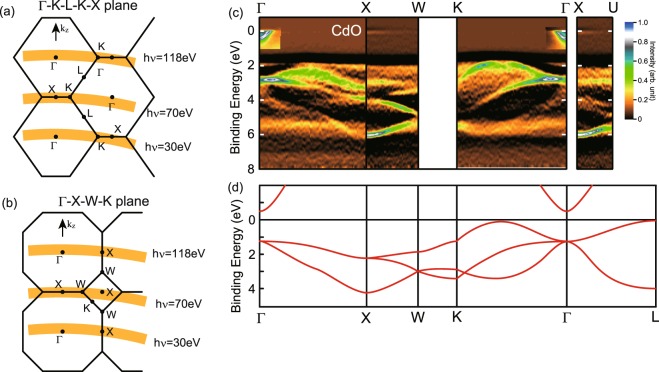
In-plane angle-resolved photoemission measurements. (**a**) The *Γ*-*K*-*L*-*K*-*X* plane. The band dispersion along *X*-*K* and *Γ*-*K* symmetry lines are measured at the photon energies of 70 and 118 eV, respectively. Conduction band features were measured at the photon energy of 30 eV. (**b**) The *Γ*-*X*-*W*-*L* plane. The band dispersions along *X*-*W* and *Γ*-*X* symmetry lines are measured at the photon energies of 70 and 118 eV. (**c**) The intensity plot of the second derivative EDC in *E*-*k* space. (**d**) The calculated band dispersions have been reproduced from ref.^16^.

Besides the normal-emission measurements, in-plane angle-resolved measurements have been performed with tuning the photon energy. As shown in Fig. 4a, the band dispersion along wave vector parallel to the surface including *Γ* point (*Γ*-*X* line) and *X* point (*X*-*W* line) can be measured using the photon energy of 118 and 70 eV, respectively. While the dispersion along *Γ*-*X* line has been measured with both 118 eV and 30 eV, the conduction band features around the *Γ* point are clearly resolved with the photon energy of 30 eV. After the azimuthal rotation of 45° within the sample surface, the band dispersion along the wave vector parallel to the surface at the *Γ* point (*Γ*-*K* line) and the *X* point (*X*-*U* line) were measured using the photon energy of 118 and 70 eV as shown in Fig. 4b. The observed band structure is summarized in Fig. 4c. The overall features of the band dispersions resemble those previously calculated (Fig. 4d)^16^. Two bands are dispersing downward from the binding energy of 2.8 eV at the *Γ* point to 4 and 6 eV at the *X* point. In addition to these bands a projected dispersion of the hole band located on *Γ*-*K* symmetry line is observed in the ARPES image measured with the photon energy of 118 eV. The lower binding energy band on *Γ*-*X* line has a degeneracy of 2 and disperses to lower and higher binding energies when the degeneracy is lifted on the line from *X* to *W* point. The band at higher binding energy on *Γ*-*X* line disperses to lower binding energy from *X* to *W* point. Three bands are distinguished in the ARPES mapping for *Γ*-*K* direction. The upper band with a maximum midway along the *Γ*-*K* line of the valence band is clearly resolved in the ARPES mapping. This is in agreement with the theoretical calculations that attribute this effect to the *p*-*d* mixing^18^. It should be noted that the projected band dispersion along the *Γ*-*L* direction, which contains the valence band maximum (VBM) position is superimposed on the ARPES mapping along the *Γ*-*K* direction, because the *Γ*-*K*-*L*-*K*-*X* plane includes the *Γ*-*L* line.

**Figure 5:**
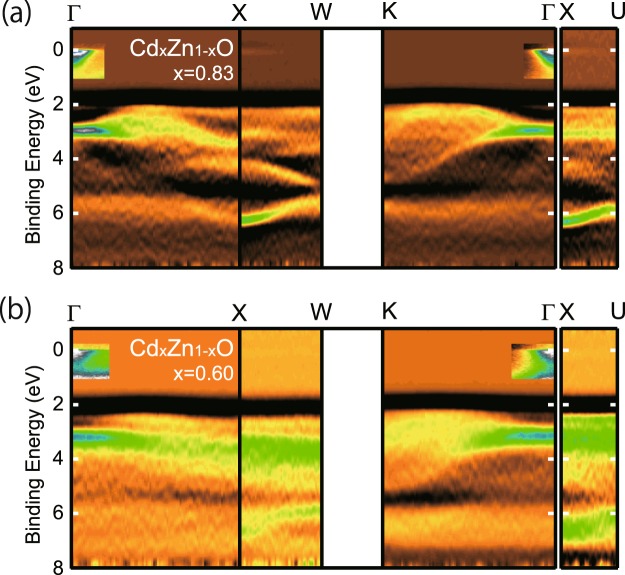
The intensity plot of the second derivative of EDCs in the energy and momentum space for Cd_0.83_Zn_0.17_O (**a**) and Cd_0.60_Zn_0.40_O (**b**).

Figure 5 shows the three dimensional band dispersion of Cd_*x*_Zn_1−*x*_O (*x* = 0.83 and 0.60) measured under similar condition as those shown in Fig. 4. Binding energies of observed bands at *Γ*, *X*, *W*, and *K* high symmetry points are well determined by ARPES measurement. The overall valence band features are similar for *x* = 0.83 and 0.60. The valence band features shift to higher binding energies with increasing Zn content, in agreement with reported XPS results^9^.

**Figure 6:**
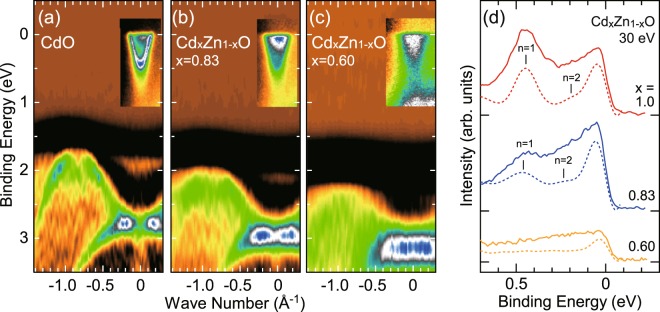
(**a**–**c**) Detailed valence and conduction band dispersions along Γ-K line measured with the photon energy of 118 and 30 eV, respectively. (**d**) Energy distribution curves (solid lines) and their second derivatives (dashed lines) of conduction region at the zone center.

Figure 6a–c show detailed valence and conduction band dispersion along the *Γ*-*K* line. Valence and conduction band features were measured with the photon energy of 118 and 30 eV, respectively. Valence bands are shown using the second derivative images of photoemission spectra. Conduction bands are the photoemission intensity images. As shown in Fig. 6, the valence band features shift to higher binding energies with increasing Zn content. VBM position at midway along the *Γ*-*K* line, where band dispersions along the *Γ*-*L* line are also projected, shows larger shift than the valence band at the zone center. Binding energies of observed bands at *Γ*, *X*, *W*, and *K* high symmetry points are summarized in Table 2 after the compensation of the amount of band bending at the surface. The decrease of the lattice constant due to the replacement of Cd site by smaller Zn atoms accounts for the increase of the band gap. In addition, the energy difference between *Γ*_15_ and VBM decreases with Zn-doping, indicating the reduced *p*-*d* hybridization with increased Zn content.

**Table 2:** Binding energy of the observed bands at *Γ*, *X*, *W*, and *K* high symmetry points and corresponding direct and indirect gap values E_g_ from ARPES measurements.

	*x* = 1.0	0.83	0.60
L_3_ (VBM)	0.91	1.24	1.49
Γ_15_	1.92	2.18	2.27
X′_5_	3.13	3.48	2.4
X′_4_	5.14	5.50	5.74
W_1_	2.51	2.72	2.88
W_3_	4.13	4.44	5.1
*n* = 2 (eV)	0.19	0.23	—
*n* = 1 (eV)	0.43	0.47	—
m^*^/m_0_ (*n* = 1)	0.14	0.17	—
CBM	0.13	0.15	<0.5
band bending (eV)	0.87	0.76	0.82
z_0_ (Å)	26	32	—
N_D_ (cm^−3^)	3.5 × 10^19^	2.0 × 10^19^	—
E_g_^ARPES^, (direct)	1.79	2.03	~2.0
E_g_^ARPES^ (indirect)	0.78	1.09	~1.2

The position of bulk CBM and the depth of surface potential and corresponding charge concentration were determined to reproduce the binging energies of quantized sub-bands with n = 1 and 2.

The spectra in Fig. 6 show conduction band features found just below the Fermi level around the zone center. The features corresponding to the quantized two-dimensional electronic states associated with the accumulation layer on the surface are clearly resolved for CdO (*x* = 1.0). The bottom of the first sub-band on CdO is located at the binding energy of 0.43 eV. Figure 6d shows the energy distribution curve (solid line) and its second derivative (dashed line) of the conduction region at the zone center. As shown in Fig. 6d, a weak trace of *n* = 2 state is also identified at 0.20 eV for *x* = 1.0. In the *x* = 0.83 sample, the first and second sub-band features are found at 0.47 and 0.23 eV, respectively. On the other hand, no quantized sub-band can be resolved for the sample with *x* = 0.60. The observed smearing out of the quantized features can be attributed to the poor crystalline quality of the *x* = 0.60 sample. While it is difficult to distinguish the bulk conduction band from the smeared sub-bands, the photoemission intensity related to conduction band states extend to about 0.5 eV. Thus, the lower bound of the conduction band minimum of *x* = 0.60 sample would be 0.5 eV below the Fermi level. From the small shift of charge neutrality level with Zn content, it is concluded that the increase of optical band gap with Zn content is mainly related to the increase of the intrinsic band gap rather than the B-M effect, which imply that the high transmission in IR region would be preserved on the alloying with Zn.

**Figure 7:**
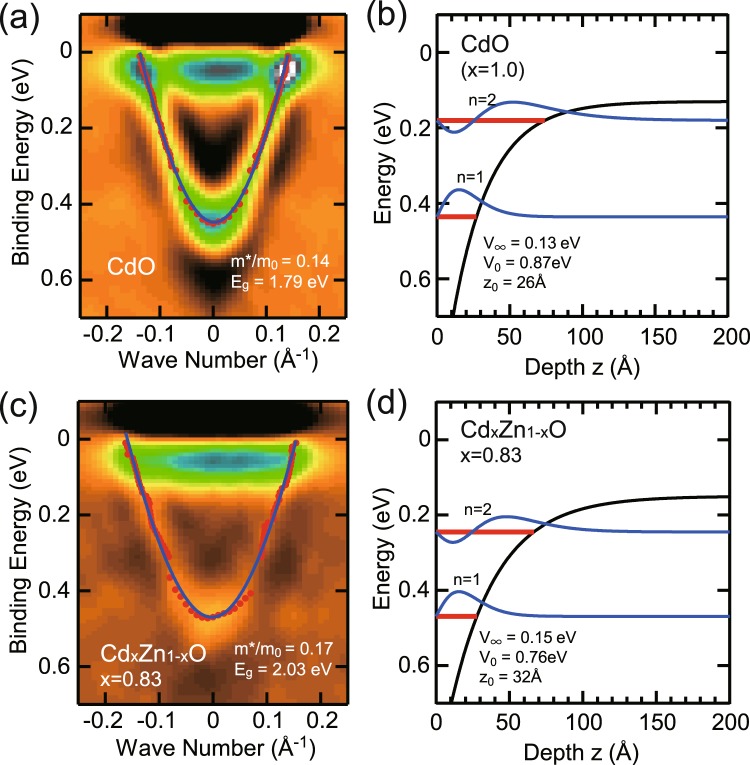
(**a**) ARPES intensity map at near Fermi level of CdO (x = 1.0) measured at *hν* = 30 eV. The conduction sub-band dispersion determined from the peak fitting to the MCD and EDC are plotted by solid circles. (**b**) The surface potential V(*z*), calculated energy levels and corresponding wave function for *x* = 1.0. (**c**) ARPES intensity map and (**d**) calculated energy levels and corresponding wave function for *x* = 0.83.

In order to see the dispersion of conduction sub-bands in detail, the second derivative images of photoemission spectra for *x* = 1.0 and 0.83 are shown in Fig. 7a,b, respectively. The in-plane band dispersion of quantized sub-bands with *n* = 1 have been determined from peak positions on energy and momentum distribution curves and are plotted using solid circles in Fig. 7a,b. Solid lines in Fig. 7a,b indicate results of least-square fittings with non-parabolic dispersion based on the Kane ***k·p*** approximation^25^ using direct band gap values of 1.79 and 2.03 eV. These band gap values have been determined for the energy separation between valence band at Γ point and conduction band minimum (CBM) determined to reproduce the subband position using a model potential as discussed below. The obtained band edge effective masses are approximately 0.14*m*_0_ and 0.17*m*_0_ for *x* = 1.0 and 0.83, respectively. The effective mass from optical measurement for the bulk conduction electrons is slightly smaller than 0.21*m*_0_^26^. The dispersion of quantized conduction band is isotropic with respect to the parallel wave-vector. Fermi wave vector of the sub-band with *n* = 1 is about 0.14 and 0.16 Å^−1^ for *x* = 1.0 and 0.83, respectively. The corresponding sheet carrier concentrations of *N*_2D_ = *k*_f_^2^/2*π* ~ 3.1 × 10^13^ and 4.1 × 10^13^ cm^−2^ are estimated using Luttinger area of Fermi surface^27^. The quantized conduction sub-band with *n* = 1 has a smaller binding energy than that reported in previous work^16^ where CdO films with the thickness of 380 nm were grown on *r*-plane sapphire using metal-organic vapor phase epitaxy and cleaned in UHV by an annealing at 600 °C, that is higher than our annealing temperature of 300 °C. This could affect the concentration of electrically active surface adsorbates which, as has been shown in refs^28,29^, are important factors determining the surface band bending.

The quantized two-dimensional conduction-band-states in the electron accumulation layer have been modeled assuming an exponential quantum well V(*z*) = −V_0_ exp(−*z*/*z*_0_), where *V*_0_ is a surface potential and *z*_0_ is a characteristic length of the potential^13^. The eigenenergies of the quantized levels are obtained by solving the one dimensional Schrodinger equation. The non-parabolicity of the conduction band is included via the energy dependence of the effective mass. The eigenenergies of quantized states are given by *E*_b_ = *ℏ*^2^*p*^2^/8 m^*^z_0_^2^, where the *p* values are given by roots of the Bessel function *J*_p_(*q*) = 0 with *q* = [8 m^*^ *V*_0_*z*_0_^2^/*ℏ*^2^]^1/2^. In our modeling, the band bending potential of *V*_0_ = 0.87 (for *x* = 1.0) and 0.76 eV (for *x* = 0.83) are adopted from measured core-level shifts shown in Fig. 2. Also the values of effective masses of 0.14*m*_0_ (for *x* = 1.0) and 0.17*m*_0_ (*x* = 0.83) from the in-plane dispersion in Fig. 7a,c were used in the calculations. The observed energies of sub-bands with *n* = 1 and 2 at the zone center have been well reproduced in the calculations with characteristic length *z*_0_ of 26 and 32 Å, and bulk CBM positions of 0.13 and 0.15 eV for *x* = 1.0 and 0.83, respectively. The determined bulk CBM position corresponds the bulk Fermi wave vector of 0.075 and 0.085 Å^−1^ for *x* = 1.0 and 0.83, respectively. The corresponding carrier concentration *N*_3D_ = *k*_f_^3^/3*π*^2^ is 1.4 × 10^19^ cm^−3^ and 2.1 × 10^19^ cm^−3^ for *x* = 1.0 and 0.83, respectively. The thickness of band bending region at surface is related to the bulk carrier concentration and roughly estimated as the Debye length of *L*_D_ = [2*V*_0_*εε*_0_/*e*^2^*N*_D_]^1/2^, where *N*_D_ is the bulk carrier density^30^. The characteristic length *z*_0_ of confinement potential roughly corresponds to *L*_D_/3. Using a static dielectric constant of *ε* = 21.9^31^, the bulk charge densities of 3.5 × 10^19^ cm^−3^ and 2.0 × 10^19^ cm^−3^ are estimated for *x* = 1.0 and 0.83, respectively. These values are consistent with those from the bulk CBM positions. Figure 7b,d show the surface potential V(*z*), calculated energy levels and corresponding wave function Ψ(*z*) = J_p_(*q* exp(−*z*/2*z*_0_)) for *x* = 1.0 and 0.83, respectively. The charge densities of first and second sub-bands are found to be localized around 19 and 48 Å below the surface. These localized states contribute to the surface electric current. Recently, it has been reported that sub-band energies and corresponding surface carrier accumulation are greatly increased by electric dipole layer with the adsorption of molecules on the surface of degenerated semiconductors^28,29^. Thus, both parental bulk-carrier-density and surface carrier distribution that greatly depends on the environment should be carefully taken into account for the exact evaluation of transport properties.

**Figure 8:**
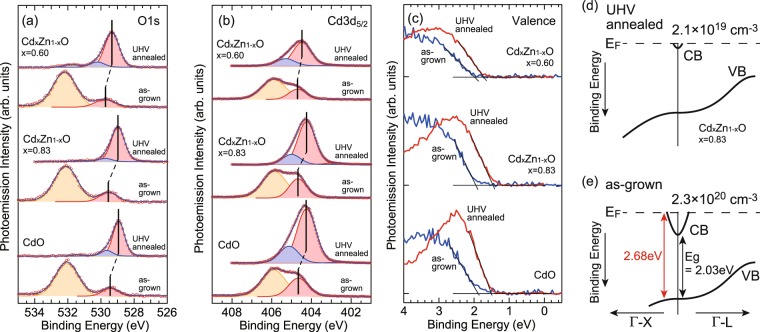
(**a**) O 1s, (**b**) Cd 3d_5/2_, and (**c**) valence band spectra of as-grown and UHV annealed samples. (**d**) Schematic diagram of bulk conduction and valence bands of annealed sample with *x* = 0.83. (**e**) Schematic band diagram of as-grown *x* = 0.83 sample.

Figure 8a–c show photoemission spectra of as-grown (before annealing) and UHV-annealed samples. O 1s and Cd 3d_5/2_ core levels were curve fitted using Voigt shape peaks. Whereas as-grown samples show large contamination components, bulk components can be well resolved. The VBM positions were determined by extrapolating linear fits for the baseline of valence band spectra. As shown in Fig. 8a–c, bulk components and VBM position of as-grown samples are located at about 0.5 eV higher binding energies than those of UHV-annealed samples. Figure 8d shows the schematic valence and conduction bands of Cd_*x*_Zn_1−*x*_O with *x* = 0.83. The bulk carrier density of UHV-annealed sample was estimated as 2.1 × 10^19^ cm^−3^ from the depth of surface potential that reproduce the energies of quantized states in the surface accumulation layer and also from the Luttinger volume of conduction band whose bottom is located at 0.15 eV below the Fermi level. Figure 8e shows the schematic band diagram of as-grown samples determined by assuming a rigid shift of valence and conduction bands to 0.5 eV higher binding energy. The Fermi wave vector of the conduction band is estimated to be about 0.19 Å^−1^. The corresponding carrier concentration is 2.3 × 10^20^ cm^−3^. The optical band gap is also estimated as about 2.7 eV. These values are in good agreement with those from optical absorption and Hall measurements. The decrease of carrier density upon the annealing have also been reported previously and described as the decrease of unintentional carriers by the removal of defects or atoms at interstitial sites^26^.

## Conclusion

We have performed angle-resolved photoemission spectroscopy (ARPES) using synchrotron radiation to determine the three-dimensional band structure of rock-salt (rs) Cd_*x*_Zn_1−*x*_O (*x* = 1.0, 0.83, and 0.60). A decrease of *p*-*d* hybridization with Zn content accounts for the larger increase of indirect band gap than that of the direct band gap. The charge neutrality level in conduction band shows small shift with Zn content. Thus, the increase of optical band gap is mainly related to the increase of the intrinsic band gap with Zn content rather than the B-M effect. Two-dimensional electronic states due to the quantization along the surface normal direction are formed in the surface accumulation layer and show non-parabolic dispersion. The binding energy of quantized two-dimensional state is well reproduced using an accumulation potential with the observed surface band bending and the characteristic width of about 30 Å.
